# Cracking AlphaFold2: Leveraging the power of artificial intelligence in undergraduate biochemistry curriculums

**DOI:** 10.1371/journal.pcbi.1012123

**Published:** 2024-06-27

**Authors:** Devon J. Boland, Nicola M. Ayres

**Affiliations:** Department of Biochemistry & Biophysics, Texas A&M University, College Station, Texas, United States of America; bioinformatics.ca, CANADA

## Abstract

AlphaFold2 is an Artificial Intelligence-based program developed to predict the 3D structure of proteins given only their amino acid sequence at atomic resolution. Due to the accuracy and efficiency at which AlphaFold2 can generate 3D structure predictions and its widespread adoption into various aspects of biochemical research, the technique of protein structure prediction should be considered for incorporation into the undergraduate biochemistry curriculum. A module for introducing AlphaFold2 into a senior-level biochemistry laboratory classroom was developed. The module’s focus was to have students predict the structures of proteins from the MPOX 22 global outbreak virus isolate genome, which had no structures elucidated at that time. The goal of this study was to both determine the impact the module had on students and to develop a framework for introducing AlphaFold2 into the undergraduate curriculum so that instructors for biochemistry courses, regardless of their background in bioinformatics, could adapt the module into their classrooms.

## Author summary

AlphaFold2 is software that combines sequence similarity and structure templating with the power of Artificial Intelligence (AI) to bridge the connection between the primary protein structure (amino acid sequence) and higher-level 3D structure (secondary, tertiary, and quaternary). AlphaFold2’s impressive and easily accessible nature makes it a bioinformatics tool that has been seeing a wide range of applications in biochemical research. Given this large-scale application, we examined whether Alphafold2 could be integrated into an undergraduate curriculum. We developed a novel module for a senior-level undergraduate biochemistry laboratory class. Our goal was to lay a solid foundation for other undergraduate instructors to be able to adapt this module to fit their classroom needs. While we implemented and ran all predictions on an internal university computing cluster, we recommend ColabFold for those instructors who do not have access to large-scale computational clusters or whose internal clusters cannot scale to their classroom sizes. We have outlined 3 metrics to be quantitatively investigated in the module to give both instructors and students metrics to evaluate model confidence. We have also included a template worksheet, lecture slides, and example scripts to enable instructors to rapidly develop a similar module. We hope that more departments and programs will integrate AlphaFold2 into their undergraduate curriculums, giving students a highly in-demand skill to prepare them for their transition into a career or graduate school.

## Introduction

The importance of structural biology in biochemistry is taught in nearly every biochemistry textbook used in biochemistry lectures. Protein structure aids researchers in various capacities, such as drug discovery, protein engineering, biotherapeutic development, and many other biotechnological applications. Typically, protein structures are elucidated using 1 or a combination of 3 techniques: x-ray crystallography [1], NMR spectroscopy [2], or cryo-electron microscopy [3]. These protein structure-solving techniques are widely practiced but are typically reserved for training in graduate school [4]. This is mainly due to the complexity and time it takes to produce results; it does not fit into a “bite”-sized module or semester class schedule. Just as effort in computer hardware and software development transformed the old days of assembling DNA sequences by hand from gel electrophoresis, there has been a substantial push to leverage computational power to solve protein 3D structures. Every other year since 1994, hundreds of research groups have competed in the Critical Assessment of Structure Prediction (CASP), which has served as the longstanding benchmark for the accuracy and measure of feasibility for predicting protein structures [5].

In 2020 at CASP14, the first ever “success” (>90% accuracy in predicting protein structure) was achieved by the artificial intelligence company DeepMind’s (now Google DeepMind) AlphaFold2 [5,6]. AlphaFold2 broke previous boundaries by employing large machine-learning models that could predict highly accurate structures for proteins with sequence homology to other proteins that already had solved structures and in proteins that exhibited little to no sequence homology to known sequences [6]. The latter directly showed machine learning’s decisive role in a global or template-free approach [7], i.e., not having a reference structure as a proverbial “blueprint” to build a model. The inherent impact on the biochemical research community that AlphaFold2 has had and continues to have cannot be understated. Recently, it has been used to solve systems so large and complex that they had previously eluded researchers using traditional methods for solving protein structures, such as the cytoplasmic ring of the nuclear pore complex [8]. AlphaFold2 can also supply virtual drug library screening structures that are in many biopharmaceutical companies’ pipelines. However, it should be noted that cautious evaluation of the final model should be taken into consideration before drawing any conclusions [9].

We developed a learning module that introduced the 3D protein structure prediction software AlphaFold2 to a senior-level biochemistry laboratory class. AlphaFold2 is an ideal bioinformatics tool since it fits the criteria of a “click-and-go” software. It is executed by running a “wrapper script” that performs all steps needed to produce a 3D protein structure, with minimal parameter optimization and coding required by the end user. The other benefit of using AlphaFold2 to introduce undergraduates to bioinformatics is that much of the knowledge needed to interpret the results of AlphaFold2 extends from a basic understanding of protein folding and structure. All of this combined makes AlphaFold2 an ideal tool to explore for introduction at the undergraduate biochemistry level.

The AlphaFold2 module was divided into two 3-h class sessions with a total of 64 undergraduate students. Students and faculty were surveyed with standardized questions adapted from the Colorado Learning Attitudes in Science Survey (CLASS) Field [10] to evaluate the module. CLASS question sets have been used and modified to assess student perception of undergraduate classwork in physics, chemistry, biology, and computer science [10–13]. To emphasize the novelty of AlphaFold2 and its unique applications, protein structures were predicted for protein sequences isolated from the MPOX 22 global outbreak USA virus isolate (ON563414.3). Students were tasked with evaluating the resulting predicted structures in both a qualitative and quantitative capacity. Finally, students were given a worksheet (S1 File) to evaluate their structure models and to help them assign functional annotations based on domain sequence homology to the NCBI Conserved Domain Database [14].

## Results

**Fig 1:**
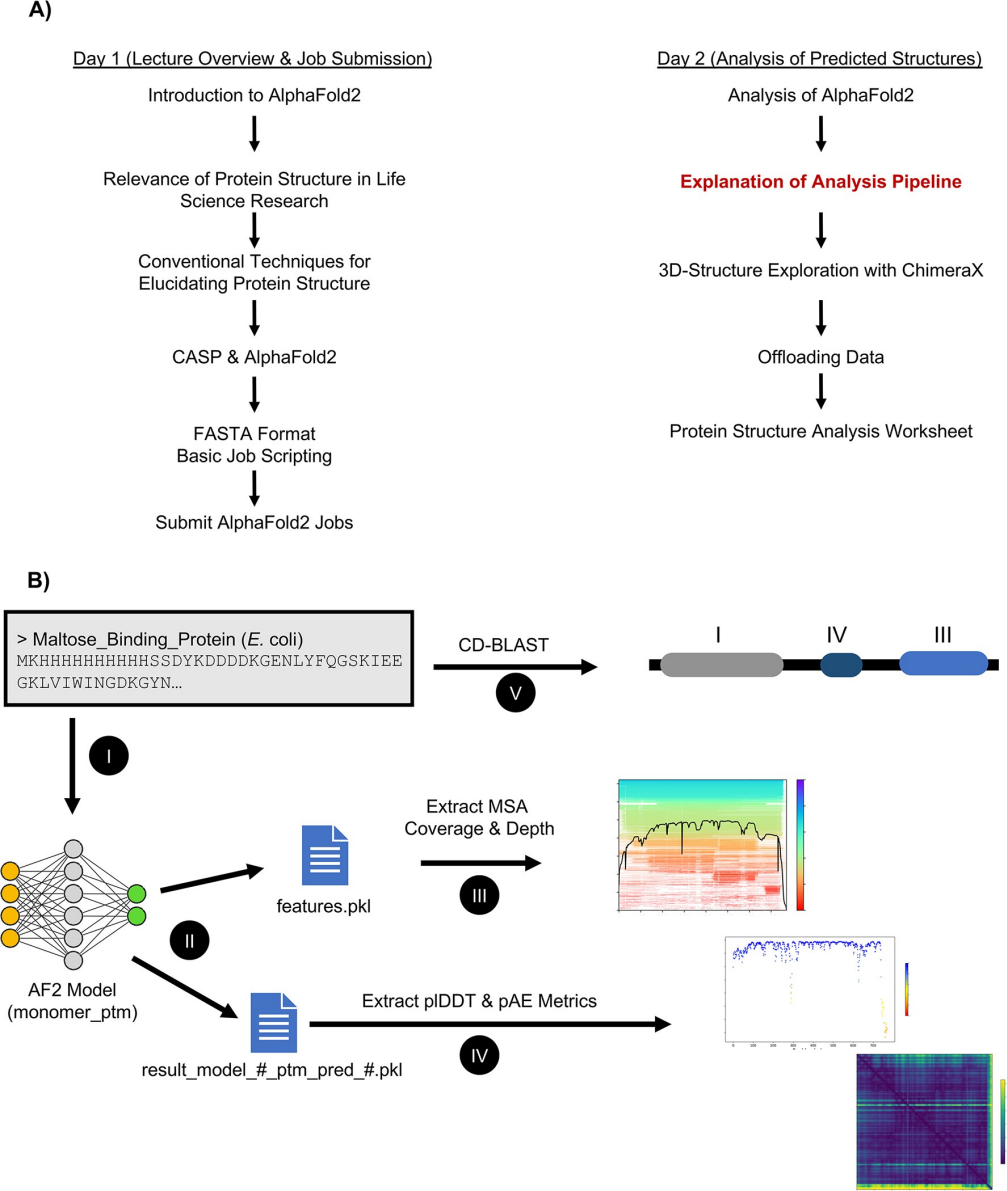
Overview of the AlphaFold2 module. (A) Simple flowchart detailing the breakdown of each lecture and class work accomplished between both days of the module. The red bolded text is expanded in (B) schematic of AlphaFold2 workflow from a student perspective. A fasta sequence file was used as input to AlphaFold2 (I), the features.pkl and model_ptm.pkl files were extracted from the AlphaFold2 output directory (II), MSA sequencing homology and depth were extracted (III), pLDDT and pAE values were extracted (IV), and finally, the structure was investigated by analysis against the conserved domain database (V).

**Fig 2:**
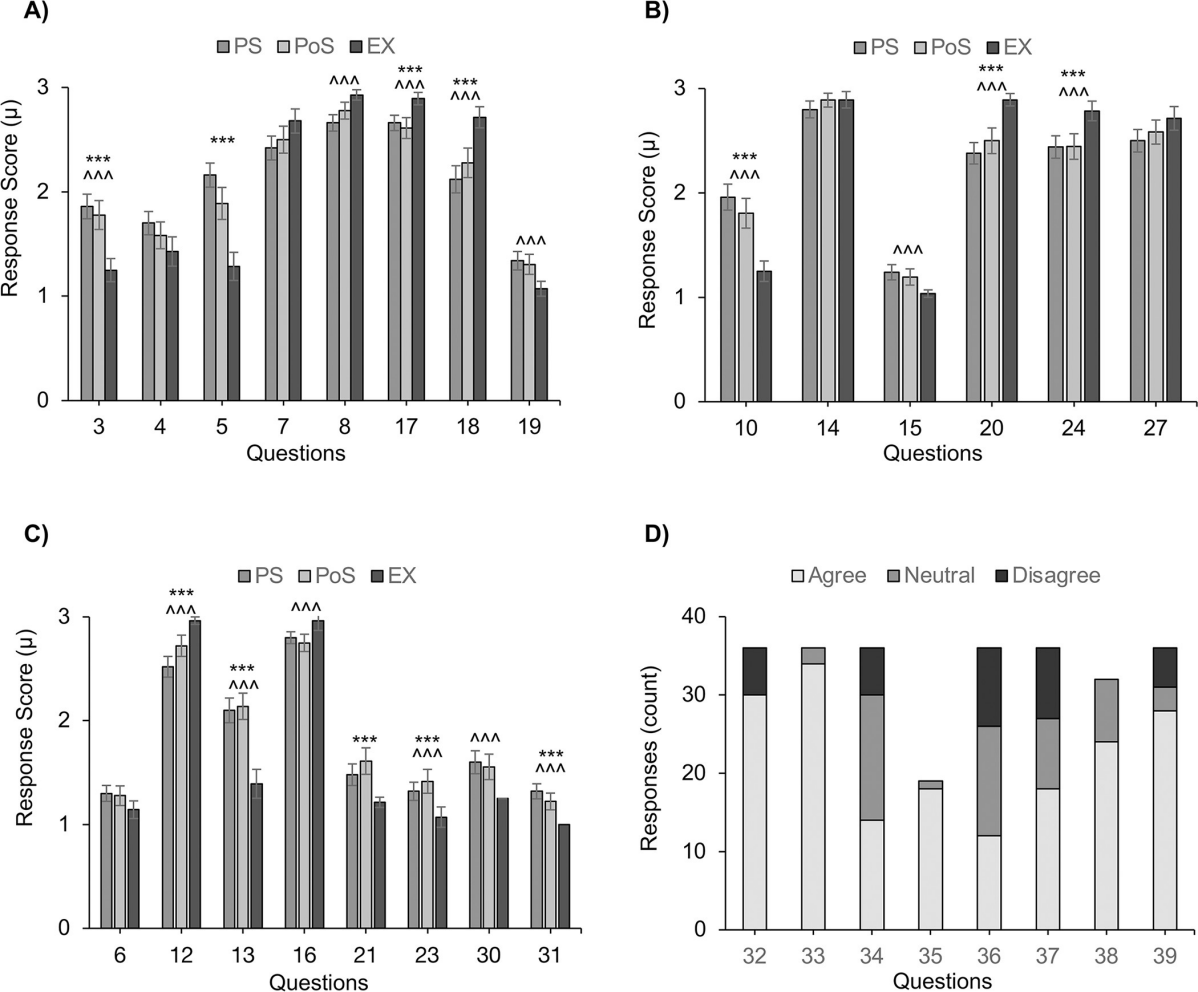
Statistical analysis of a subset of the CLASS survey responses in pre-module students (PS), post-module students (PoS), and experts (EX). (A–C) Clustered bar charts of average response scores for 22 of the 31 CLASS questions based on the 3-point Likert scoring system. Error bars represent standard error values. *** and ^^^ denote statistical significance from computed *t* test statistics based on *p*-values (*p* < 0.05) for PS to EX and PoS to EX, respectively. PS, PoS, and EX are colored dark gray, light gray, and black, respectively. (D) Response sums for the 8 additional post-module questions. Responses are color-coordinated light gray, dark gray, and black for “Agree,” “Neutral,” and “Disagree,” respectively. Note that question 35 does not have the same number of responses, as students were only asked to respond under special conditions. A complete list of the questions and analysis of all the survey data can be found in the Supporting information.

Students and experts were surveyed for their responses to standardized CLASS questions meant to probe attitudes to learning biochemistry in the classroom and how a module focused on implementing a novel tool could affect this perception (Table 1 and Figs 1 and S1–S3 and S2 File). Experts included faculty, staff, and postdoctoral researchers with a doctoral degree in the Department of Biochemistry at Texas A&M University. The experts agreed (as defined in the Methods section, >66.6% agreement, see S3 Fig) for all questions. Statistical analysis was carried out between the 3 sets of response groups: pre-module students (PS), post-module students (PoS), and experts (EX). Since not all participants in the PS survey completed the PoS survey, direct statistical analysis between these student participant groups was avoided to not introduce systemic bias. In most cases, student responses started to approach experts’ answers after the module (Figs 2A–2C and S7 File).

**Table 1:** Survey statements organized by CLASS category. Statements were closely adapted from a previous adaptation of the CLASS-BIO set [12]. Similarly, statements were changed by replacing the words “biology” and “molecular biology” with “biochemistry”.

Statement	CLASS Category
1,2,8,10,12,14,15,24	Personal Interest (Enjoyment)
2,10,12,13,16,21,30	Real World Connection
3,5,18,28	Problem Solving: Synthesis & Application
6,17	Problem Solving: Strategies
7,10,17,20,24,27,28	Problem Solving: Effort
13,20	Problem Solving: Reasoning
9,11,16,19,29,31	Conceptual Connections
4,22,23,26	Uncategorized

CLASS, Colorado Learning Attitudes about Science Survey.

This module was formulated to show an alternative approach to students, highlighting that AlphaFold2 was a tool that could produce biologically relevant 3D structures of proteins when traditional structure elucidation methods were not feasible. Student post-module (PoS) responses were statistically indistinguishable from expert responses (EX) in questions 8, 15, and 19 (Fig 2A and 2B), while pre-module student responses (PS) were statistically distinguishable. Question 8 in the survey probed the opinions of the students on how they approach a problem after failing at it the first time. This module was a clear, practical lesson in how traditional methods can fail in the laboratory, but another approach could produce experimental results. Student opinions shifted to be statistically indistinguishable from the experts’ opinions.

**Fig 3:**
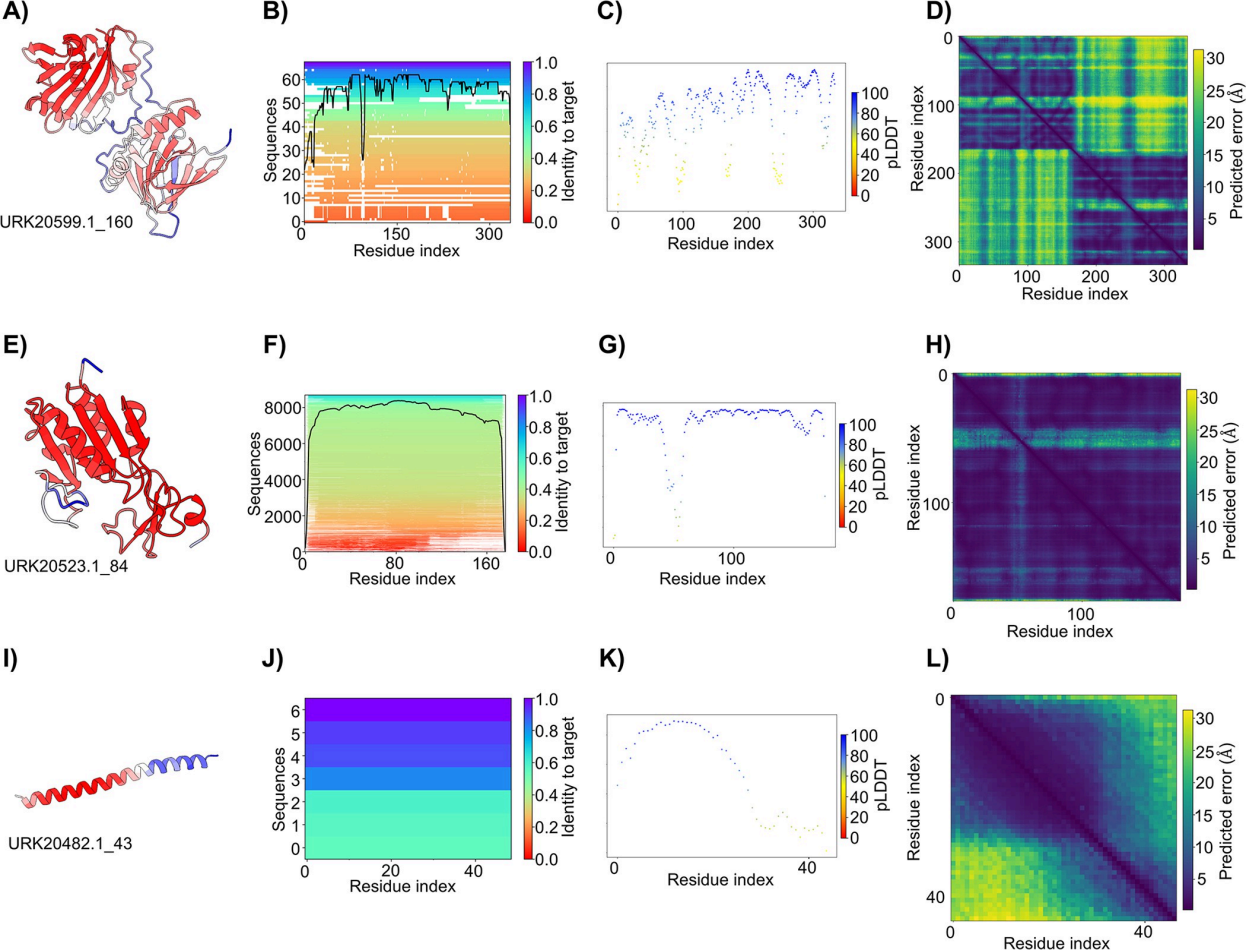
Example of data extracted and analyzed from the AlphaFold2 models. (A, E, I) Three protein sequences from the MPOX 22 virus USA isolate had structures predicted in and rendered with ChimeraX chain coloring, indicating pLDDT value at each residue with blue to red indicating low to high pLDDT. (B, F, J) MSA depth and homology plots extracted from the features.pkl file from the AlphaFold2 output directories. Each graph corresponds to the accompanying protein structure. The number of homologous sequences in the MSA was plotted along the y-axis and colored based on sequence identity. The black line indicates the total number of sequences that aligned at a given target residue. (C, G, K) Scatter plots of the pLDDT value at each residue in the target sequence, color-coded by pLDDT value at the residue. (D, H, L) 2D heatmaps of the calculated pAE for each protein structure in A, E, and I. These structures and data were chosen as a representative sample of the class data to show the wide variety of predicted structures in terms of overall folding, local, and relative model confidence.

Another facet of this module was that, depending on the protein sequence being analyzed, the predicted structure from AlphaFold2 could vary rapidly in model confidence metrics (Fig 3A–3L) depending on sequence homology to other known proteins. Question 15 probed student opinions on whether learning biochemistry relies on memorizing facts and definitions. PS responses were statistically different from EX responses, while PoS responses more closely resembled EX responses and were not statistically different. While only a minor shift in the average response, it speaks to the fact that laboratory classes typically use widely applicable techniques and protocols, but in real applications, these can often be met with confounded outcomes. This would require the students to rely on their understanding of biochemical principles and begin to explain why their data deviated from the expected result.

Student opinions were questioned on whether biochemistry is related to their everyday life (Question 19); it had a similar outcome as Question 15. Before the module, PS responses were statistically different from EX responses, while post-module PoS responses were not statistically different. A probable explanation for this difference in response is that the protein sequences used in this module were from the recent (when conducting this study) 2022 MPOX outbreak USA virus isolate. Next, students stuck on biochemistry-related questions were asked if there was little to no chance that they could figure it out independently (Question 21). Interestingly, student responses diverged (Fig 2C) from expert opinions post-module. Not only did the PoS responses differ from the EX-response, but they were statistically different. This marks the only survey question where post-module student responses did not converge with expert opinions. Again, this likely stems from the complexity of biochemistry and math introduced in this module.

Most interestingly was the idea that applying methods or techniques used to understand one biochemical problem to another problem would require the issues to be connected in some way, which differed drastically between students and experts (Question 11). Student opinions remained the same after the module compared to experts (S1–S4 Figs), with a mean response of 2.24 and 2.25, respectively, compared to the expert’s mean response of 1.28. This suggests that structural proteomics and the complex problem of elucidating a protein’s 3D structure from the amino acid sequence opened the students to a complexity they likely had not previously seen in a classroom setting.

A particularly relevant question regarding introducing bioinformatics into the undergraduate curriculum is the dependency of mathematics in understanding biochemical topics (Question 26). A previous study on implementing a bioinformatics-based module into a molecular biology class showed that students agreed with experts only after the module [15]. Interestingly, student responses before and after the module agreed with the experts and were not statistically distinguishable from the experts’ responses (S4 Fig). This may be due to the apparent difference in the structure of a molecular biology versus biochemistry laboratory class and the mistaken belief that modern bioinformatics arose from the need for analyzing next-generation sequence data [16].

Post-module students were additionally surveyed with a custom set of questions (S2 File) meant to probe the effectiveness of this module and students’ perception of it (Fig 3D). About 83% (30/36) of PoS responders indicated that their curiosity about bioinformatics had increased as a direct result of this module. Additionally, approximately 94% (34/36) PoS responders indicated that the module had also introduced them to the use of computer science in biochemistry. This was an important goal when designing the module; typically in undergraduate biochemistry curriculums, there is a lack of emphasis on how computer science has played and will continue to play a significant role in the development and execution of biochemical research [17].

Question 35 asked students if they were not enjoying computational data analysis. Of 19 out of 36 that responded, approximately 94% (18/19) indicated that this module gave them a good idea of how biochemists handle large protein datasets. We wanted to show students what happens to proteins after their sequences and structures are deposited into public databases such as NCBI and the Protein Data Bank. The last 4 questions of the additional post-module survey questions, 36 to 39, tried to elucidate how practical the module was for increasing student interest in bioinformatics concerning biochemistry. Only approximately 33% (12/36) of responders indicated they were interested in pursuing additional bioinformatic-related coursework due to the module. This number is not surprising as a majority, approximately 95%, of the students indicated they planned to attend medical school or work in a different field. However, 50% (18/36) indicated they were interested in learning more about high-performance research computing and how it could apply to their research and career goals. Even more interesting was that most responders indicated that, while they prefer traditional wet-lab/field-work experimentation to work that requires computationally intensive analyses (approximately 66%, 24/36), they understood that their future career in biochemistry would rely on basic knowledge of bioinformatics/computational biochemistry (approximately 77%, 28/36).

## Discussion

The field of biochemical research is becoming more reliant on bioinformatics, and it is our duty as educators to prepare coursework that reflects current techniques and methodology in undergraduate curriculums. Our novel AlphaFold2 module was designed to fit within an existing biochemistry laboratory or classroom format, with little to no bioinformatic framework needed for implementation. We chose AlphaFold2 based on its open-source nature [6], readily available implementations [18], and that the final output is a 3D-viewable file that can be interpreted through basic biochemical knowledge of protein structure and function. We concentrated on building a two-part module that focused on teaching the basics of protein-structure prediction, introducing students to the Protein Data Bank repository, having students predict the structure of an unclassified protein and extract meta-data such as sequence homology, pLDDT, and predicted aligned error (pAE) as metrics for evaluation in model confidence. We found that the approximately 64 students responded well to the module and enjoyed learning about this newer technique that has emerged in the field of biochemical research. We surveyed students before and after the module and experts independently with altered CLASS questions. We additionally analyzed students post-module with custom questions probing their resulting interest in bioinformatics from this module. Our surveys found that introducing a “hands-on” module centered around a current global health-related problem, the MPOX ‘22 outbreak, reinforced the applicability of the techniques and theories taught in the classroom in their everyday lives. Students were introduced to a complex problem facing the larger biochemical research community of predicting protein 3D structures. They were taught how to use AlphaFold2 to probe for these structures and how to evaluate the model confidence based on 3 useful metrics. While the students, mostly graduating seniors, did not seem ready to dive into bioinformatics-heavy coursework moving forward in their careers, they remained impressed by AlphaFold2. They understood the dependence biochemical research has on bioinformatics and the benefit it could bring to their research. Student engagement in the module was overwhelmingly positive, and its impact on future academic coursework selection was likely masked by the large proportion of students in the course who were graduating at the end of the semester. However, any conclusions drawn on this module’s effect on future bioinformatic coursework would be better served with surveys with a refined set of post-module questions accounting for the graduation dates of the students.

Based on our pilot study with this module, we recommend a few changes that may increase the learning potential and enjoyment for the students. First, we recommend increasing the number of proteins that each student predicts. In our pilot, each student was only assigned a single protein sequence to predict a structure for and to analyze. Many studies have shown that repetition correlates to increased retention [19]. Due to the “unpredictable” nature of the predicted structures and the level of confidence one could have in the resulting model, the comfort level of the students for viewing and analyzing protein 3D structures would likely increase by having the students repeat the post-structure prediction analysis, MSA, pLDDT, and pAE inspections, combined with increased time viewing and manipulating structures in a molecular viewing software. The learning potential would also likely improve by increasing the amount of time dedicated to the module. By devoting more class time to the module, students could spend more effort thoroughly analyzing their predicted protein structures. Further analyses could be added, such as molecular docking, to simulate a typical in silico workflow for screening drug targets. Lastly, in future adoptions of this module in any fashion, the authors recommend substituting the protein functional annotation by the NCBI Conserved Domain tool [14] with InterPro [20].

It is important to point out that in this case, the course Biochemical Techniques I is a junior/senior level biochemistry techniques laboratory meant to reinforce with bench work what students have learned in their previous biochemistry lecture courses. The laboratory is constructed to closely follow a set of published research papers from former faculty members of the department, wherein students mutate, express, and purify ribonuclease Sa and assay it for its catalytic and thermostable activity. Within the 15-week semester that the course is offered, this experimental journey takes approximately 11 weeks. The remaining 3 weeks are used to incorporate additional content, while the final week is reserved for a final and practical examination. This includes guest lecturers covering topics such as Cryo-EM, protein folding, and mass-peptide fingerprint analysis. Inclusion of this module did not require instructors to modify the course content as it became part of the additional material. However, we are aware that whenever instructors consider introducing new content into the classroom, there is always the question of what must be sacrificed to meet term timelines. If instructors do not have additional time to allot to this module or cannot sacrifice already prepared content, we suggest modifying this module in a way that includes the use of AlphaFold2 as a method of analysis such as having students utilize AlphaFold2 to predict the 3D structure of a protein they are analyzing in the lab. In the case of proteins that already have 3D structures elucidated, students could potentially evaluate the predicted protein structure complex between their protein of interest and a target protein-ligand using the AlphaFold2 multimer model.

For educators who do not have access to a high-performance computer cluster on their campus, we highly recommend utilizing ColabFold [18]. ColabFold offers the entire Alphafold2 package and runs on Google’s free cloud computing resources. The developers behind ColabFold replaced the initial MSA construction step with a significantly faster search algorithm. This results in final structure predictions typically ranging between 30 min to 1 h in our own test cases. While ColabFold results in quicker predictions, instructors should be aware that there are some limitations. These include predictions aborting due to limited RAM which typically occurs when dealing with larger proteins/complexes, and/or proteins with large levels of sequence homology among the databases that AlphaFold2 queries. We highly recommend that instructors test ColabFold on a representative subset of their proteins before giving them to their students. With these free resources offered, users can predict structures for small, simple proteins on the time scale of approximately 1 h with built-in metrics for analyzing MSA depth, pLDDT, and pAE for the target protein sequence. Students can then download their results directly to their Google Drive for downstream analysis.

### Supplemental information

Sample code scripts, a worksheet template, and lecture slides along with additional linked information are available as a GitHub repository (https://github.com/DevonJBoland/AF2-for-undergraduate-curricula) to facilitate easier adoption by instructors. The authors will continue to update and support this repository as they continue to adapt and iterate over the module.

## Methods

### Ethics statement

The Texas A&M Institutional Review Board approved surveys of students and experts under Texas A&M IRB: IRB2023-0423M. Surveys were conducted in such a way as to provide total anonymity to students and experts. No personal identification information was collected from any survey participant and surveys were administered by teaching assistants not participating in the study. During the response collection period, any responder could withdraw from the study through the survey instruments, and their submissions would be deleted from the collection.

### Design of the AlphaFold2 module

The Biochemical Techniques I laboratory class at Texas A&M University is a 15-week class in the spring of each academic year. The class consists primarily of senior biochemistry and genetics majors and has 2 sections with a combined total of 64 students that meet twice weekly for 2 h and 50 min each class period. The class is structured to have the students follow the experiments in a series of publications that examine the effect of charge on protein activity and stability, using the enzyme ribonuclease Sa [21–24]. The students perform site-directed mutagenesis to make mutants with altered charge at physiological pH. They induce protein expression, purify the protein using chromatography, analyze the purity using SDS-PAGE, and confirm the identity of the protein using MALDI-TOF tandem mass spectroscopy. Finally, they determine if the mutation has altered the enzyme kinetics or protein stability of ribonuclease Sa and use ChimeraX for molecular modeling. Over the semester, the students write up their experiments in the style of a research publication.

The last 3 to 4 weeks of the class are typically reserved for guest lecturers, who cover various topics of current biochemical research. The instructor allocated 2 class periods in the last 4 weeks of the course to give the students experience with AlphaFold2. The 2 sessions were designed to achieve separate goals (Fig 1A). The first session was used to introduce the students to AlphaFold2 (https://github.com/DevonJBoland/AF2-for-undergraduate-curricula/, Lecture 1), including a mini-review on the basics of protein structure, the role of protein 3D structure in life science research, experimental techniques for solving protein structures, the history of predicting protein 3D structure including biennial CASP meetings, and ending on the basics of bioinformatic file formats and job scripting for running AlphaFold2 on the Texas A&M University High-Performance Research Computing group (HPRC) Grace cluster [25]. The second-class session was held 1 week after the first. It included a lecture (https://github.com/DevonJBoland/AF2-for-undergraduate-curricula/, Lecture 2) on the best practices to evaluate the predicted model confidence using previously published metrics from the predicted structure research community. The students were then shown how to download their data and explore the predicted 3D structures with the open-source software ChimeraX [26]. Before the incorporation of this module, students previously had a molecular modeling module built into the course, wherein students would download the 3D structure of ribonuclease Sa from the RCSB Protein Data Bank (PDB) and view and inspect the structure following a guided worksheet in ChimeraX. In this case, this module was removed, and students followed a new guided worksheet (S1 File). This worksheet was designed to not only introduce first-time users to the basic controls of ChimeraX, but also the finer points of structural analysis such as measuring bond distances, and visualizing protein structure surface area. Finally, the students were tasked with using CD-BLAST [14] and multiple sequence alignments to suggest a function for their protein sequence.

### Prediction of MPOX ‘22 protein structures

The MPOX ‘22 genome assembly had 190 genes annotated at the time of this study; the corresponding protein amino acid sequences were downloaded from NCBI (GenBank: ON563414.3). These protein fasta sequences were randomly assigned so that each student received a single, unique protein sequence. The students then used AlphaFold2 (v2.1.1) to predict the protein’s 3D structure. Due to a lack of default parameters, all arguments had to be declared in the execution of the AlphaFold2 wrapper script (S3 File). An entire database (—db_preset = full_db) search was performed, and the monomer_ptm (—model = monomer_ptm) model was used so that predicted aligned error metrics would be computed during model prediction. The Texas A&M HPRC Grace cluster was used to host and run the protein structure prediction jobs. AlphaFold2 jobs can run on NVIDIA GPUs during the final steps of model refinement to accelerate run time. To avoid the jobs of about 64 students being bottlenecked by the 4 GPU nodes on the Grace cluster that meet AlphaFold2 requirements, all jobs were run on CPU nodes, significantly increasing run-time. To accommodate this, jobs were given 24-CPU cores, 160 GB of RAM, and a total wall time of 1 day to run before the job scheduler killed them. Typically, structure predictions finished in approximately 4 h. The top-rated model was downloaded and investigated by students during the second session of the module.

### Analysis of homologous sequence depth and coverage of target proteins

To analyze the model confidence, 3 separate metrics were investigated by each student. A custom script generated the appropriate graphs from the AlphaFold2-generated output. The first step in the AlphaFold2 wrapper script performs an extensive search of many metagenomic databases and builds a multiple sequence alignment for the target sequence. This MSA is mainly influential in the final predicted model’s accuracy compared to experimentally elucidated structures [6,27]. MSA depth and homology were inspected by running a custom script to extract the number and percent similarity of homologous sequences from the “features.pkl” file in the AlphaFold2 output directory. To facilitate the ease of deploying this custom script and to prevent students from having to set up virtual environments or install additional software, the scripts were uploaded and formatted to work with Google Colab Jupyter notebooks (S4 File). This script produces a graph (Fig 3B, 3F and 3J) with the residue index of the target sequence on the x-axis and the number of homologous sequences included in the MSA on the y-axis. Each index in the homologous sequence is colored with a heat map corresponding to sequence identity where 0.0 corresponds to no similarity and 1.0 is identical. These values are extracted from the jackhammer [28] search performed during the MSA generation in AlphaFold2. Lastly, a black line is traced onto the graph, indicating the density of sequence alignment at a particular index of the target sequence. This density is defined as the number of sequences that were identified as homologous at a given residue in the protein amino acid sequence (the higher this value, the better). Previous studies of AlphaFold2 suggest that an optimal threshold for a “good” model is to have approximately 100 homologous sequences at a given residue for a target protein [29]. While there is no method for generating a definitive cut-off, our large sampling of protein prediction models and our analyses (Fig 3) suggests that fewer than 30 homologous sequences generate “ambiguous” structural motifs and, in some cases (Fig 3I–3L) may be due to fragmented gene annotation in the genome assembly.

### Analysis of pLDDT values for local model confidence

Traditionally, the local distance difference test (LDDT) has been widely used to evaluate predicted structures [30]. LDDT is a superposition-free score, meaning it does not require a reference structure to compute the distance differences of all atoms in each model. Additionally, the LDDT can score the validity of a given stereochemical plausibility. While the computational methods employed for calculating LDDT can be overly complex to teach in an undergraduate biochemistry laboratory class, the general idea behind what LDDT informs about a given predicted model is not. The concept of LDDT was introduced to the students in the first session of the module and presented as a quantitative value assessing the “plausibility” of a given set of atoms in a local 3D space. The higher this value is, the more confidence one can have in the model mimicking an experimentally derived structure. The pLDDT values are also stored in the result_model_#_ptm_pred_#.pkl file in the AlphaFold2 output directory and are extracted and plotted using alphaPICKLE [31]. This script generates a scatter plot (Fig 3C, 3G and 3K) where each point in the graph represents the average pLDDT of all atoms in each residue of the target sequence. For increased visual inspection, the dots are colored based on the pLDDT value. Generally, “community” set thresholds are included in the FAQ section on the AlphaFold2 predicted structure database hosted by EMBL-EBI [32]. It is stated that predicted structure regions can fall into 4 categories ranked by their pLDDT scores. Regions with pLDDT > 90 are expected to model with high accuracy and, in test cases, appear to mimic experimentally derived structures [6,32]. Regions with a score of 70 < pLDDT > 90 have a highly accurate backbone prediction but may deviate in stereochemical position in side-chain residues. Regions with a score of 50 < pLDDT > 70 are considered low confidence and should be marked as unreliable, or the user should exhibit caution when designing any experiments based on the structure. Any region scoring pLDDT < 50 usually displays a randomly disordered coil structure and cannot/should not be interpreted. This unstructured coil could arise from a highly dynamic region that could not be captured in traditional experimental structure elucidation methods or maybe highly intrinsic in physiological conditions.

### Analysis of predicted aligned error for relative model confidence

While pLDDT is a valuable metric for understanding local model accuracy at the atomic level, predicted protein structures must also be evaluated on where residues are placed relative to the whole model. Another helpful confidence metric that AlphaFold2 computes during the model refinement is the pAE. This metric is calculated by overlaying the predicted model with itself and calculating the positional error between 2 residues across the alignment with the C⍺, N, and C atoms [32]. When this value is low, the model is predicted to have high confidence in the relative position between the residues. When expanding the view from a single amino acid pair to entire regions and domains within a predicted structure, one can attribute relative confidence to the model’s 3D position. This becomes a helpful metric for insight into multi-domain proteins or even protein complexes predicted by AlphaFold2 [27]. alphaPICKLE [31] was also used to extract and plot the pAE metrics stored in the output directory of AlphaFold2. It is important to note that to have the pAE metrics computed, AlphaFold2’s model parameter (—model) must be set to “monomer_ptm”; otherwise, the pAE metrics will not be computed during model prediction.

### Evaluation of AlphaFold2 module with survey analysis

Survey instruments were created and administered through Google Forms (https://www.google.com/forms/). Three individual surveys were conducted for pre-module student responses (PS), post-module student responses (PoS), and expert responses (EX). Experts were defined as any faculty, staff, or postdoctoral researchers within the department who had a doctoral degree in either biology, chemistry, or biochemistry. All 3 surveys contained the same set of 31 CLASS questions. The PS and EX surveys were identical, with a total of 31 questions, while the PoS survey had the same 31 questions, with an additional set of 8 questions (S2, S5, and S6 Files and S1–S3 Figs).

Pretreatment of the collected responses was conducted in a two-step fashion. Question 26 of the CLASS questions served as a control question that asked the responders to fill out a particular answer to remove any respondent’s answers that had not carefully read the question sets. None of the participants (51 pre-module students, 37 post-module students, and 29 experts) failed to answer the question correctly. The second pretreatment step was to remove any responder’s answers if they failed to answer all the questions. Four responders failed to fill out all the questions in the PS, PoS, and EX, with 2, 1, and 1 responder from each group, respectively.

To compare student responses to expert opinions, questions were only considered for downstream analysis if expert opinions reached a consensus defined as >66% of expert answers falling under a single possible response of “Agree,” “Neutral,” or “Disagree.” All 31 questions reached a consensus among the 28 filtered expert responders. Statistical analysis was conducted on the responses of each group’s remaining 31 CLASS questions using the Student’s *T* test. Responses were assigned point values following the 3-point Likert system [33] with 1 point for “Disagree,” 2 for “Neutral,” and 3 points for “Agree.” From there, each question per response group had mean and standard error calculated, *t* test statistic, and *p*-value computed using the SciPy (S4 Fig) [34] Python statistical package through a custom script (S7 File). PS and PoS responses were compared to expert responses and were considered statistically different with a *p*-value threshold of 0.05 (*p* < 0.05) (S5 File). To gauge any effect the module had on students’ interest in pursuing bioinformatics, 8 additional questions were given and administered to students only after the module finished (S2 File). The questions focused on whether students would pursue additional bioinformatics coursework and their perception of the potential impact bioinformatics could have on their research/career.

## Supporting information

S1 Fig Stacked bar chart showing the total number of responses for each of 31 CLASS questions for pre-module student surveys by categorical response. “Agree” is colored dark gray, “Neutral” is colored light gray, and “Disagree” is colored black.
(TIF)

S2 Fig Stacked bar chart showing the total number of responses for each of 31 CLASS questions for post-module student surveys by categorical response. “Agree” is colored dark gray, “Neutral” is colored light gray, and “Disagree” is colored black.
(TIF)

S3 Fig Stacked bar chart showing the total number of responses for each of 31 CLASS questions for expert surveys by categorical response. “Agree” is colored dark gray, “Neutral” is colored light gray, and “Disagree” is colored black.
(TIF)

S4 Fig Clustered bar chart of average response score and standard error for each of the 31 CLASS questions used in the survey. Survey groups are color-coded as pre-module students (black), post-module students (light gray), and “experts (dark gray).
(TIF)

S1 File Example of the worksheet given to students on the second day of the module to guide them through the analysis of their predicted protein structures. (DOCX)

S2 File Word document containing the modified CLASS questions and custom post-module student questions used in the surveys. (DOCX)

S3 File Example code script showing the parameters used in all AlphaFold2 predictions within the module. (TXT)

S4 File Jupyter notebook for the extraction and plotting of the MSA information from the AlphaFold2 output. This notebook was run within the free Google Collaboratory application.
(IPYNB)

S5 File Excel file containing the tally for each categorical response from the surveys used in calculating the quantitative values from the 3-point Likert system. (XLSX)

S6 File Student t test statistic scores and corresponding p-values for the comparisons between the pre-module and post-module student survey responses. (XLSX)

S7 File Python code used to perform the post-survey statistical analysis. (TXT)
