# Supplementary figures and images for: Cracking AlphaFold2: Leveraging the power of artificial intelligence in undergraduate biochemistry curriculums
Source: PLoS Comput Biol. 2024 Jun 27;20(6):e1012123. doi: 10.1371/journal.pcbi.1012123 (PMC11210786; doi:10.1371/journal.pcbi.1012123)

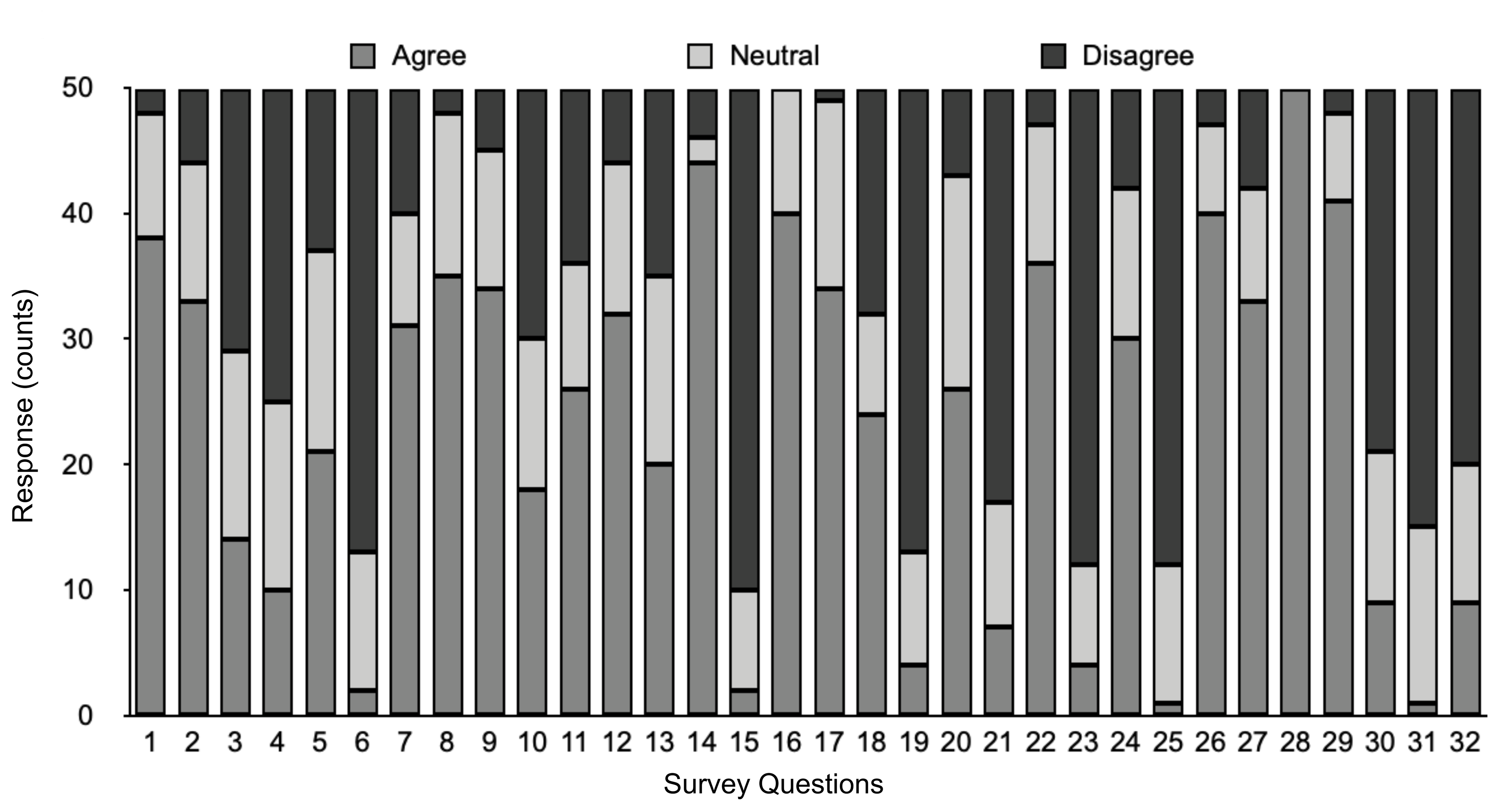

Supplement: S1 Fig — “Agree” is colored dark gray, “Neutral” is colored light gray, and “Disagree” is colored black. (TIF) [file pcbi.1012123.s001.tif]

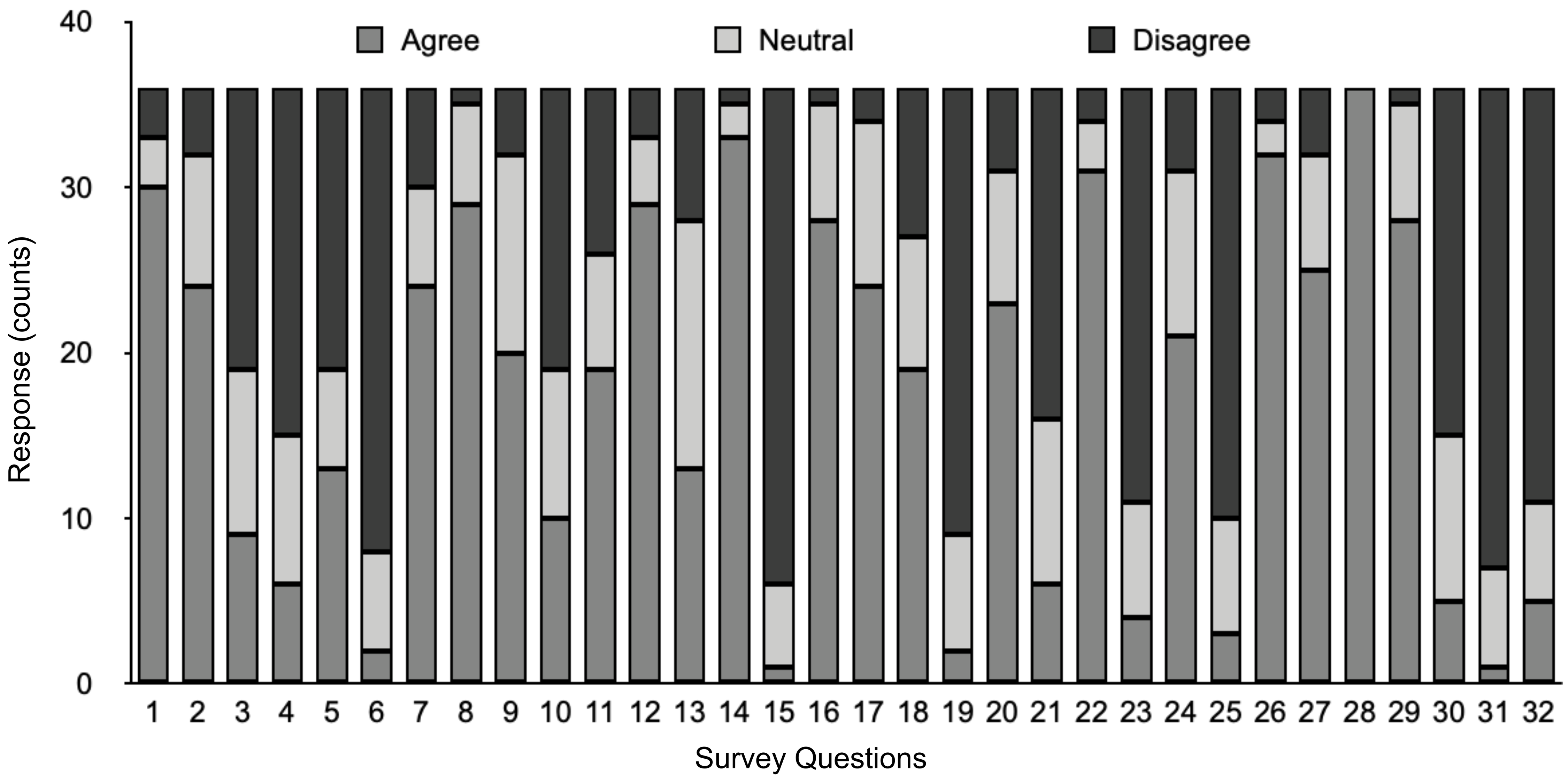

Supplement: S2 Fig — “Agree” is colored dark gray, “Neutral” is colored light gray, and “Disagree” is colored black. (TIF) [file pcbi.1012123.s002.tif]

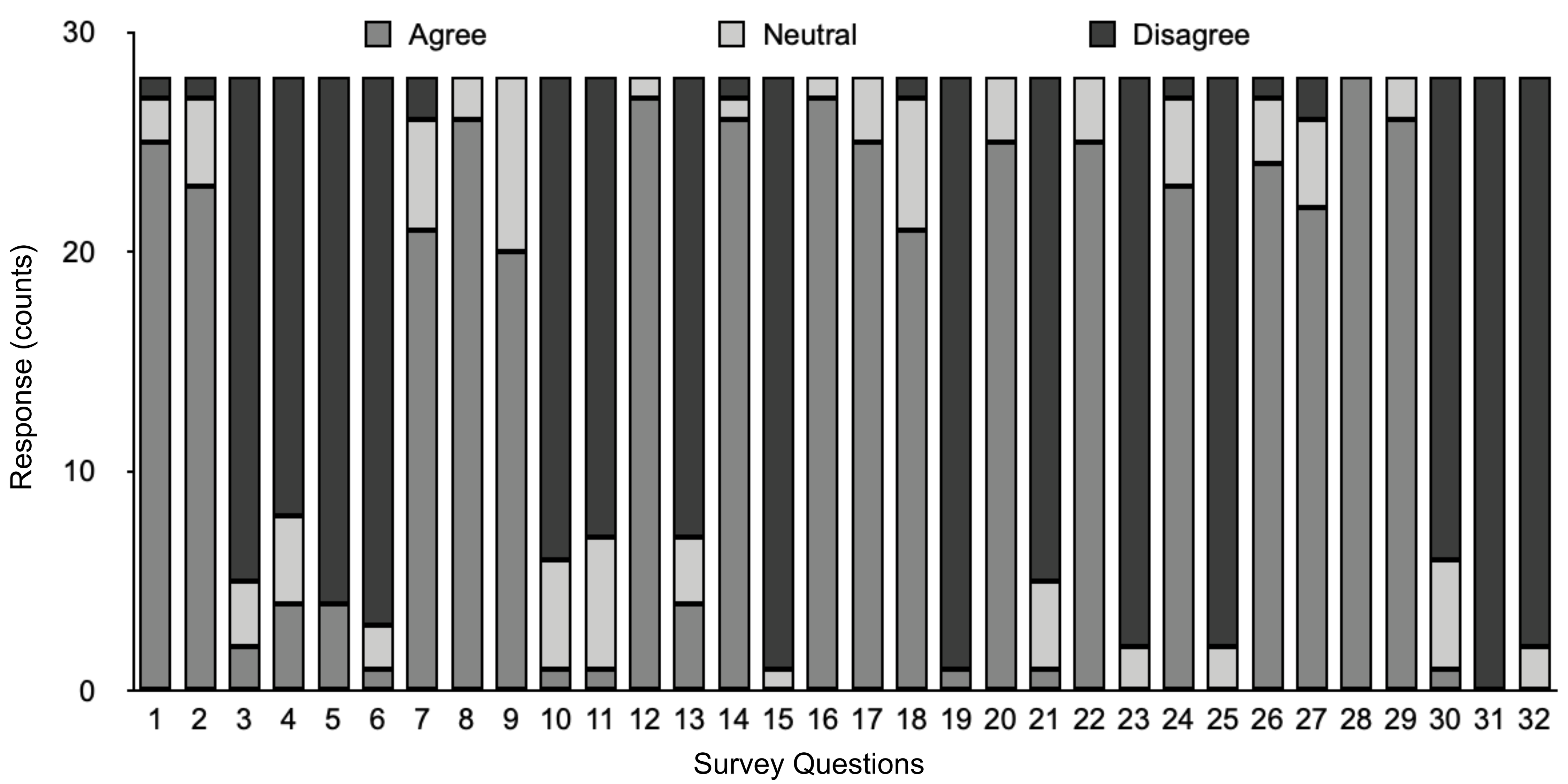

Supplement: S3 Fig — “Agree” is colored dark gray, “Neutral” is colored light gray, and “Disagree” is colored black. (TIF) [file pcbi.1012123.s003.tif]

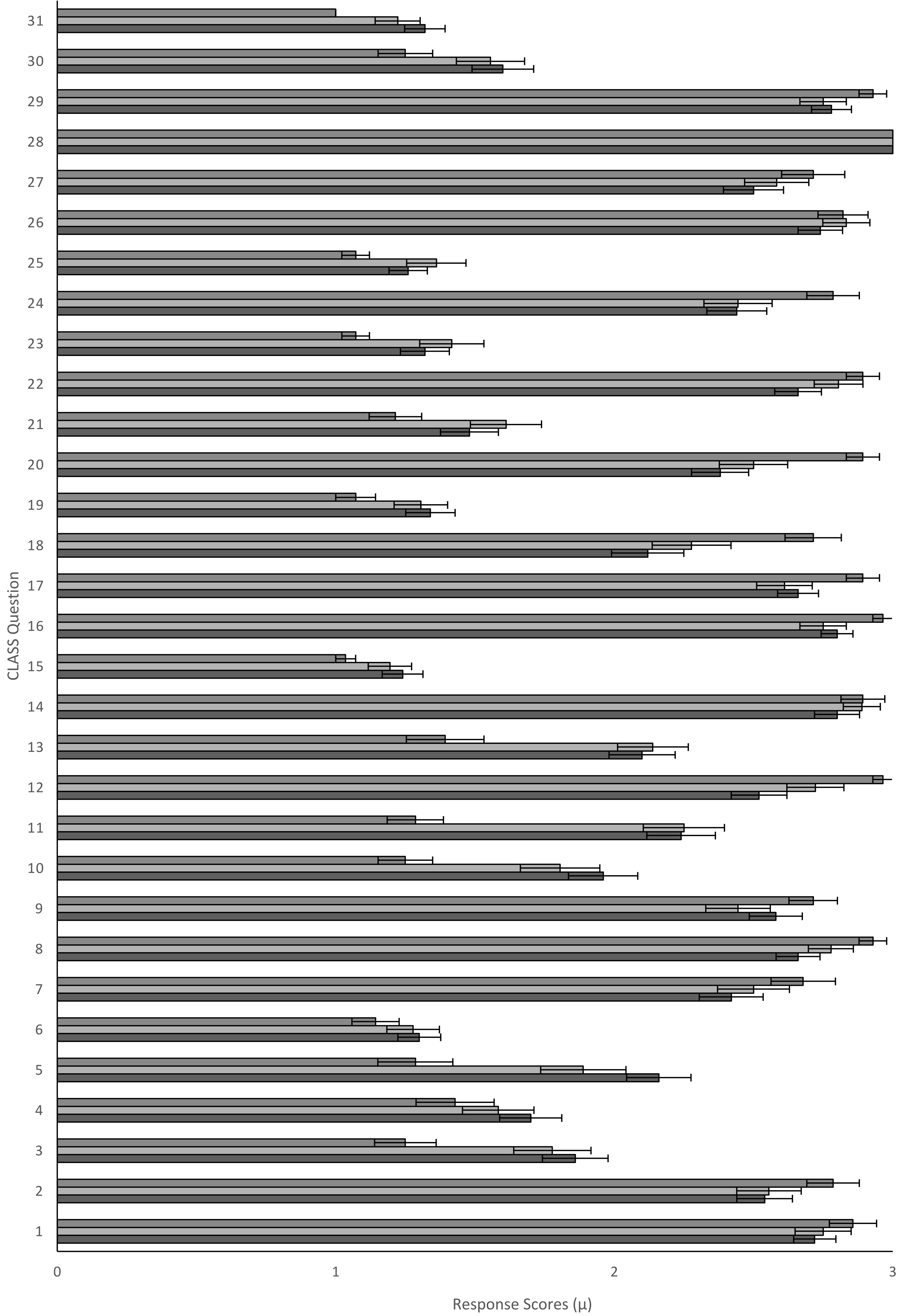

Supplement: S4 Fig — Survey groups are color-coded as pre-module students (black), post-module students (light gray), and “experts (dark gray). (TIF) [file pcbi.1012123.s004.tif]
