# Supplementary material for: Cracking AlphaFold2: Leveraging the power of artificial intelligence in undergraduate biochemistry curriculums
Source: PLoS Comput Biol. 2024 Jun 27;20(6):e1012123. doi: 10.1371/journal.pcbi.1012123 (PMC11210786; doi:10.1371/journal.pcbi.1012123)
Supplement: S1 File — (DOCX) [file pcbi.1012123.s005.docx]

**AlphaFold2 Worksheet, BICH 414 2023 (8 pts.)
Structure Analysis & Annotation**

Before we can analyze your protein structure, we need software that can render the 3D structure for us. We will use ChimeraX, developed at UCSF. It a free, open-source software that can be installed on any operation software (except Chromebooks).

Go to their website <https://www.cgl.ucsf.edu/chimerax/download.html> and download the ChimeraX application for your computer. The website should autodetect your OS and tell you which installer to download. Install the application like you would any other. Once you are finished right, click on your ranked_0.pdb file and select open with, then choose ChimeraX.

You should see a window that looks something like this:

Move on to the rest of the worksheet; remember to refer to the slides and ask any questions if you have them as you work through this worksheet.

1. View the predicted structure. You can rotate and zoom in and out on the structure using your mouse or trackpad. Let’s color the structure by the pLDDT score assigned by AlphaFold2. Remember that the higher this value (red color), the more confidence the model has, and vice versa for the blue color. AlphaFold2 stores the pLDDT score instead of the β-factor score typically obtained from experimental methods (X-ray crystallography). Click the menu “Molecule Display” and click the b-factor button.
2. Describe the overall protein structure that you observe (2-3 sentences). Does your model have well defined 3°/2° structure? Or does it have many random coils? Are there small domains that are well-defined, etc.?
3. We need to prepare some information about the structures and sequences to deposit these predicted structures into a public database. You will use a widely implemented web tool to analyze your protein sequence for amino acid composition, molecular weight, and other useful parameters. Navigate to the [Swiss-prot](https://web.expasy.org/protparam/) tool website and past your amino acid sequence from the fasta file provided to you and click submit. Fill in the below table with the information ExPasy returns.

| **Number of AA’s** | **Molecular Weight** | **Theoretical pI** | **# of positively charged AA’s** | **# of negatively charged AA’s** |
| --- | --- | --- | --- | --- |
|  |  |  |  |  |

1. Whenever we study a protein of interest, we often ask, where is this protein located in the cell? Many programs can perform a peptide analysis and determine the likely location of the protein in a cellular environment such as membrane-associated, cytosol, nucleus, etc. Navigate to the [Phobius](https://phobius.sbc.su.se/index.html) website, submit your fasta sequence, and download and save the graph to upload with all your data later. Briefly detail what the analysis suggests. Where does Phobius predict your protein to be located? Is there a transmembrane domain anywhere?

Remember that AlphaFold2 requires that our target protein have homologous sequences to have the best chance of building a confident model. We will inspect the MSA coverage that AlphaFold2 identified for our protein. We also want to check the level of confidence AF2 had in the final refined model. We also want to look at the pLDDT and pAE data as well.

At the end of the job script you submitted to Grace, a line runs a Python script called “AlphaPickle.” It is a small Python script that will extract and plot all this data for us (How convenient!). You should see a couple of PNG files in your output directory on Grace. Download them and look at them, answering the following questions:

1. Inspect the MSA coverage graph. Give a brief description of the coverage. Did we achieve the desired range across the protein sequence? Are there any gaps? Look at the protein structure in ChimeraX; if there are any areas with gaps in sequence coverage, what do they look like in the 3D structure? Is this what we expect?
2. Inspect the pLDDT plot. Give a brief description of the score across the protein sequence. Which portions of your protein structure would be reasonable for experimental design? (Remember the threshold range for pLDDT, check the slides if you forgot).
3. Inspect the pAE plot. How many domains are identified in your pAE graph (look at your protein structure to determine this)? Describe the overall confidence of the placement of each domain about other domains in the structure.

Using AlphaFold2 to predict 3D protein structure is the first step in any biochemical investigation. Other bioinformatic tools at our disposal can aid in understanding the relationship between the structure and function of a protein of interest. Due to the number of proteins studied, there is a good chance that your protein will have characterized homolog (a protein with a very similar sequence and often similar structure and function). We can use the homology theory at the protein level to infer the protein's function you modeled.

We will use BLAST (Basic Local Alignment Search Tool) to probe our sequences for potential functions. Luckily, we do not need a supercomputer for this. Navigate to NCBI’s BLAST [web portal](https://blast.ncbi.nlm.nih.gov/Blast.cgi). You will see four different buttons. Select Protein BLAST, as we want to submit a protein amino acid sequence to align with other amino acid sequences. You will be taken to another screen; paste your amino acid sequence into the query box. Leave all options at the default values and select submit. This analysis may take a few minutes to run (the NCBI databases are huge). While we wait, we will also take advantage of another blast tool called Conserved Domain BLAST (CD-BLAST) which will identify functional domains in our protein sequence. Navigate to the CD-BLAST [web page](https://www.ncbi.nlm.nih.gov/Structure/cdd/wrpsb.cgi). Paste your amino acid sequence and click submit. Once both have finished, answer the following two questions:

1. The Protein BLAST results should populate a table detailing the top homologous sequences to your protein of interest. If we look through the large alignment files output from AlphaFold2, we likely find these exact sequences there too. The NCBI website and tool will allow us to look up the complete sequences from the database. Fill out the table below with the top five results from your Protein BLAST.

| **Nearest Homolog** | **E-value** | **% Identity** | **Coverage Length** | **Accession ID** |
| --- | --- | --- | --- | --- |
|  |  |  |  |  |
|  |  |  |  |  |
|  |  |  |  |  |
|  |  |  |  |  |
|  |  |  |  |  |

1. The CD-BLAST results should be done as well. Like Question #8, fill the table below with the domains identified from the domain blast. (If your protein has more than the domains below, only include the first five)

| **Domain Name** | **Accession ID** | **Interval** | **E-value** |
| --- | --- | --- | --- |
|  |  |  |  |
|  |  |  |  |
|  |  |  |  |
|  |  |  |  |
|  |  |  |  |

1. With all the analysis you have done with your protein, compile all of this data into a two to three-sentence summary of your protein. Include what the predicted function of your protein is and list any expected co-factors, ligands, or substrates.
