# Supplementary material for: Cracking AlphaFold2: Leveraging the power of artificial intelligence in undergraduate biochemistry curriculums
Source: PLoS Comput Biol. 2024 Jun 27;20(6):e1012123. doi: 10.1371/journal.pcbi.1012123 (PMC11210786; doi:10.1371/journal.pcbi.1012123)
Supplement: S2 File — (DOCX) [file pcbi.1012123.s006.docx]

**Modified CLASS Questions**

1. My curiosity about the living world led me to study biochemistry.

2. I think about the biochemistry I experience in everyday life.

3. After I study a topic in biochemistry and feel that I understand it, I have difficulty applying

that information to answer questions on the same topic.

4. Knowledge in biochemistry consists of many disconnected topics.

5. When I am answering a biochemistry question, I find it difficult to put what I know into my

own words.

6. I do not expect the rules of biochemical principles to help my understanding of the ideas.

7. To understand biochemistry, I sometimes think about my personal experiences and relate

them to the topic being analyzed.

8. If I get stuck on answering a biochemistry question on my first try, I usually try to figure out

a different way that works.

9. I want to study biochemistry because I want to make a contribution to society.

10. If I don’t remember a particular approach needed for a question on an exam, there’s

nothing much I can do  to come up with it.

11. If I want to apply a method or idea used for understanding one biochemical problem to

another problem, the problems must involve very similar situations.

12. I enjoy figuring out answers to biochemistry questions.

13. It is important for the government to approve new scientific ideas before they can be

widely accepted.

14. Learning biochemistry changes my ideas about how the natural world works.

15. To learn biochemistry, I only need to memorize facts and definitions.

16. Reasoning skills used to understand biochemistry can be helpful to my everyday life.

17. It is a valuable use of my time to study the fundamental experiments behind

biochemical ideas.

18. If I had plenty of time, I would take a biochemistry class outside of my major requirements

just for fun.

19. The subject of biochemistry has little relation to what I experience in the real world.

20. There are times I think about or solve a biochemistry question in more than one way to

help my understanding.

21. If I get stuck on a biochemistry question, there is no chance I'll figure it out on my own.

22. When studying biochemistry, I relate the important information to what I already know

rather than just memorizing it the way it is presented.

23. There is usually only one correct approach to solving a biochemistry problem.

24. When I am not pressed for time, I will continue to work on a biochemistry problem until I

understand why something works the way it does.

25. Learning biochemistry that is not directly relevant to or applicable to human health is not

worth my time.

26. Mathematical skills are important for understanding biochemistry.

27. I enjoy explaining biochemical ideas that I learn about to my friends.

28. We use this statement to discard the survey of people who are not reading the

questions. Please select agree  for this question to preserve your

answers.

29. The general public misunderstands many biochemical ideas.

30. I do not spend more than a few minutes stuck on a biochemistry question before giving up

or seeking help from someone else.

31. Biochemical principles are just to be memorized.

32. For me, biochemistry is primarily about learning known facts as opposed to investigating

the unknown.

**Student-only Post-Module questions:**

33. My curiosity about learning more bioinformatics has increased as a result of the module

used in class.

33. The module provided new insights to me about the uses of computer sciences in biochemistry.

34. I found the computer work during this module downright enjoyable.

35. Only answer if you don’t particularly enjoy computational analyses : Although I don’t particularly enjoy computational analyses I think that this module

gave me a good idea of what scientists do with genome-size data sets.

36. As a result of this module I am now considering taking a (or another) bioinformatic course in the future.

37. As a result of this module, I am interested in learning more about High Performance Computing, and how it can aid in my research and career goals.

38. I much prefer wet-lab or field-work experimentation to projects that require lots of

computational analysis.

39. Basic knowledge of bioinformatics/computational biochemistry is likely

important for my future career (whether I like it or not).
